# Fuzzy Logic-Based System for Identifying the Severity of Diabetic Macular Edema from OCT B-Scan Images Using DRIL, HRF, and Cystoids

**DOI:** 10.3390/diagnostics13152550

**Published:** 2023-07-31

**Authors:** Aditya Tripathi, Preetham Kumar, Akshat Tulsani, Pavithra Kodiyalbail Chakrapani, Geetha Maiya, Sulatha V. Bhandary, Veena Mayya, Sameena Pathan, Raghavendra Achar, U. Rajendra Acharya

**Affiliations:** 1 Department of Information & Communication Technology, Manipal Institute of Technology, Manipal Academy of Higher Education, Manipal 576104, India; 2 Department of Computer Science and Engineering, Manipal Institute of Technology, Manipal Academy of Higher Education, Manipal 576104, India; 3 Department of Ophthalmology, Kasturba Medical College, Manipal Academy of Higher Education, Manipal 576104, India; 4 School of Mathematics, Physics and Computing, University of Southern Queensland, Springfield Central, QLD 4300, Australia

**Keywords:** DME, fuzzy engine, healthcare, clinical decision support system, computer vision, semantic segmentation, image classification

## Abstract

Diabetic Macular Edema (DME) is a severe ocular complication commonly found in patients with diabetes. The condition can precipitate a significant drop in VA and, in extreme cases, may result in irreversible vision loss. Optical Coherence Tomography (OCT), a technique that yields high-resolution retinal images, is often employed by clinicians to assess the extent of DME in patients. However, the manual interpretation of OCT B-scan images for DME identification and severity grading can be error-prone, with false negatives potentially resulting in serious repercussions. In this paper, we investigate an Artificial Intelligence (AI) driven system that offers an end-to-end automated model, designed to accurately determine DME severity using OCT B-Scan images. This model operates by extracting specific biomarkers such as Disorganization of Retinal Inner Layers (DRIL), Hyper Reflective Foci (HRF), and cystoids from the OCT image, which are then utilized to ascertain DME severity. The rules guiding the fuzzy logic engine are derived from contemporary research in the field of DME and its association with various biomarkers evident in the OCT image. The proposed model demonstrates high efficacy, identifying images with DRIL with 93.3% accuracy and successfully segmenting HRF and cystoids from OCT images with dice similarity coefficients of 91.30% and 95.07% respectively. This study presents a comprehensive system capable of accurately grading DME severity using OCT B-scan images, serving as a potentially invaluable tool in the clinical assessment and treatment of DME.

## 1. Introduction

**Figure 1:**
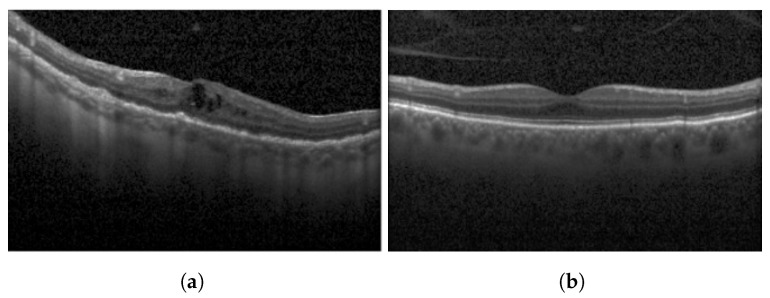
OCT B-scan image: (**a**) DME, and (**b**) Normal.

Diabetic Macular Edema (DME) is an ocular condition predominantly afflicting individuals diagnosed with diabetes. Characterized by an accumulation of intraretinal fluid within the inner and outer plexiform layers, DME triggers retinal thickening and progressively impairs visual acuity (VA), potentially culminating in irreversible vision loss. To detect this condition, clinicians typically employ Optical Coherence Tomography (OCT), a technique capable of producing high-resolution imagery of the eye. Certain parameters, referred to as biomarkers within the OCT image, serve as key indicators of DME. These include Central Subfield Thickness (CST), Disorganization of Retinal Inner Layers (DRIL), cystoid spaces, Ellipsoid Zone (EZ), and Hyperreflective Focii (HRF), all of which display a strong correlation with DME [1]. DRIL denotes an inability to distinguish the boundaries between the Inner Nuclear Layer (INL), Outer Plexiform Layer (OPL), and the Ganglion Cell Layer-Inner Plexiform Layer (GCL-IPL) complex within an OCT image. HRF, on the other hand, represents small, round lesions possessing a reflectivity equal to, or exceeding that of the Retinal Pigment Epithelium (RPE) [2]. At present, clinicians rely on these biomarkers within OCT images (refer to Figure 1) to determine the presence of DME. However, being a manual process, it is not immune to errors. False negatives can lead to an unchecked progression of the disease, resulting in a permanent impact on the patient’s VA. Therefore, a timely and accurate identification of DME is imperative for the effective management of this disease.

Several attempts have been made over the years to develop artificial intelligence (AI)-based decision support systems (DSS) for the treatment of ocular disorders by utilizing medical images and discerning the efficacy of deep learning-based models [3,4,5,6,7,8,9,10]. Given the necessity for real-world adaptability, the consensus veers towards a transparent, explainable decision rendered by DSS as more desirable compared to an opaque, albeit highly accurate, decision. Our investigation delves into an AI-oriented system that utilizes OCT B-Scan images of patients to develop a comprehensive, automated model aimed at determining the severity of DME. The cornerstone of this system is fuzzy logic, a mathematical paradigm adept at handling uncertainty and ambiguity. The inherent variability in OCT images is accommodated by fuzzy logic, allowing the system to make decisions based on fuzzy sets and linguistic rules. Our system extracts features from different biomarkers associated with DME severity, namely DRIL, HRFs, and cystoids, from the OCT image. By examining the existence and characteristics of these features in OCT B-Scan images, the fuzzy logic-based system aims to offer an objective and automated evaluation of DME severity. This can aid clinicians in making precise diagnoses, monitoring disease progression, and determining suitable treatment strategies for DME patients.

## 2. Related Work

Recently, various computer vision-related tasks have been applied in the domain of DME. Due to a limited dataset of OCT B-scan images for classifying numerous diseases, Generative Adversarial Networks (GANs) have been employed to generate additional OCT B-scan images. This helps in training diverse classification networks to detect diseases such as Age-Related Macular Degeneration (AMD), DME, and Chronic Neovascularization (CNV) [11]. He et al. [12] utilized GANs to segment retinal layers from OCT B-scan images. Similarly, Smitha and Jidesh [13] proposed an end-to-end model, leveraging GANs, to segment retinal layers from OCT B-scan images, further classifying them as either normal or disease-afflicted.

Suciu et al. [14] elaborated on the laboratory biomarkers employed to discern the severity of DME. These biomarkers include obesity, age, gender, and vascular risk factors. The average Body Mass Index (BMI) of DME patients has been observed to fall between 29 and 30. High blood pressure has also been linked to increased DME severity. Markan et al. [15] attempted to correlate cholesterol levels with DME. Their study revealed that DME severity shares a positive correlation with serum levels of total cholesterol, yet displays a negative correlation with serum High-Density Lipoprotein (HDL) cholesterol levels.

Klein et al. [16] emphasized the risks associated with using laboratory biomarkers, primarily their invasive nature and limited application in evaluating DME. An alternative approach is to assess DME severity using imagery biomarkers. With the advent of OCT, which provides high-resolution cross-sectional images of the neurosensory retina, the identification of imagery biomarkers has become significantly more accessible and reliable. The subsequent sections provide a comprehensive discussion on various imagery biomarkers employed to aid in the identification of DME and its severity.

### 2.1. Disorganization of Retinal Inner Layers (DRIL)

There are not many studies done on computer-based detection of DRIL or the quantification of DRIL extent in OCT B-Scan images. Sun et al. [17] acknowledged DRIL as a crucial biomarker for detecting DME. In another study, Babiuch et al. [18] have attempted to draw an association between DRIL and VA at baseline and after treatment in cases of Retinal Vein Occlusion (RVO). In this investigation, patients underwent Anti-Vascular Endothelial Growth Factor (AVF) therapy. The authors concluded that DRIL’s presence witnessed a diminishing trend throughout the treatment period.

### 2.2. Hyperreflective Foci

Schlegl et al. [19] proposed an enhanced version of the ResUnet model designed for segmenting HRFs from OCT B-scan images. Their model was trained on Cirrus’ and Spectralis’ OCT datasets, resulting in dice similarity coefficient (DSC) of 65.26% and 63.49% on the two datasets, respectively. On a similar note, Xie et al. [20] devised a segmentation-based model for detecting HRF in Spectral-Domain OCT (SD-OCT) images. Their approach, which combined U-Net and image enhancement techniques, yielded a DSC of 70.73%, a precision rate of 72.68%, and a recall rate of 68.89%.

### 2.3. Cystoid Spaces

Liu et al. [21] designed an algorithm for segmenting Cystoid Macular Edema (CME) using omnidirectional wave operators on OCT images. The algorithm incorporated several steps: denoising the image, applying contrast stretching to enhance it, and subsequently utilizing the wave operator after extracting the region of interest. Their study achieved a DSC of 81.1% and a recall rate of 75.0%. In a similar vein, Venhuizen et al. [22] developed a deep learning algorithm specifically for segmenting intraretinal cystoid fluid from SD-OCT images. This approach yielded a DSC of 75.4%.

Our study is mainly driven by several key motivations:

- There is a lack of research based on developing distinct machine learning models for detecting each individual biomarker.
- There is an absence of comprehensive research that integrates all biomarkers to provide a consolidated understanding of DME.
- There is a need to establish a foundational process for detecting the severity of DME using a machine learning-based approach.
- This research aims to set a benchmark for the overall prognosis of DME, utilizing biomarkers to help ascertain the severity of DME.
- Employing a fuzzy logic-based approach in the detection of DME severity is also a key motivation, as recent studies suggest that fuzzy logic has demonstrated noteworthy performance in detecting diseases [23,24,25].

## 3. Materials and Methods

The primary objective of this study is to predict the severity of DME using the OCT B-Scan of the patient. This is achieved by detecting and extracting three biomarkers (DRIL, HRFs, and cystoid spaces) from the OCT images. These biomarkers represent the severity of the disease, which is encapsulated in the form of fuzzy rules. For instance, certain locations of HRFs in the OCT image correspond to high reflectivity, thus indicating a higher severity of DME. Alongside this, numerous other rules are applied to the remaining biomarkers to ascertain the severity of the disease. A comprehensive explanation of these fuzzy rules can be found in Section 3.3.

**Figure 2:**
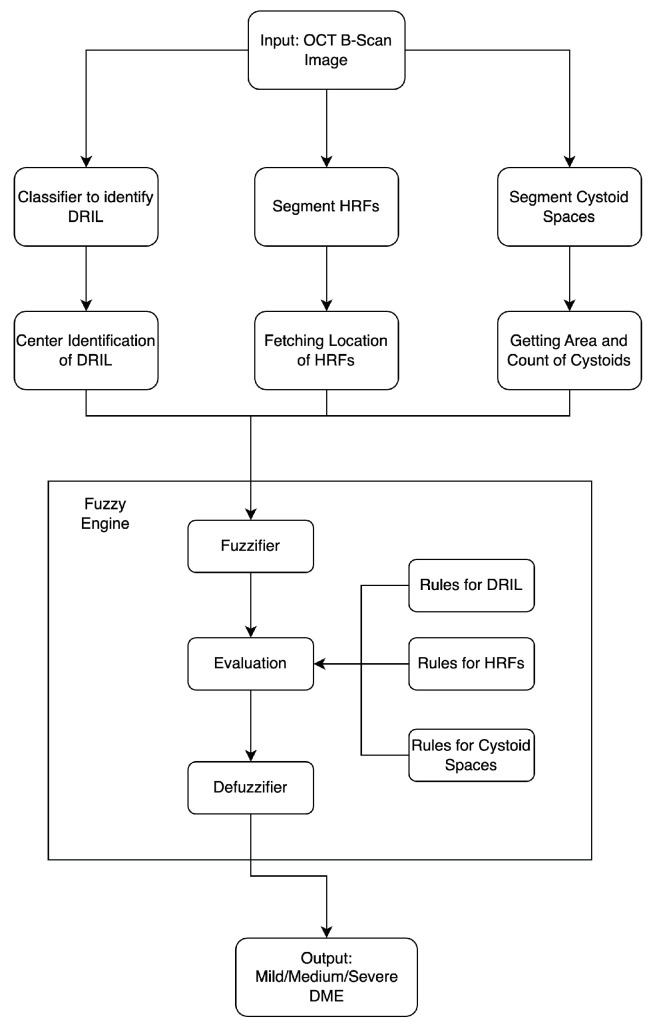
Flowchart depicting the workflow of the entire model.

The methodology adopted in this study comprises three stages: the detection of biomarkers, the generation of insights from each biomarker, and the application of these insights along with fuzzy rules to finally determine the severity of DME in the patient (refer to Figure 2). The output of this model is the predicted severity of DME based on the input OCT B-Scan image of the patient. Each stage is thoroughly detailed in the subsequent subsections.

### 3.1. Detecting Biomarkers

The initial step involves the detection of biomarkers from the OCT B-Scan image. As delineated by Endo et al. [26], numerous biomarkers such as DRIL, EZ, FAZ, inner hyperreflective foci count, height of intraretinal fluid, and others are detectable. Furthermore, the study also demonstrates DRIL has a positive correlation with both the EZ and FAZ. As such, the presence of DRIL alone can serve as an indicator of the condition of EZ and FAZ. Similarly, Arthi et al. [2] finds a positive correlation between HRF and SRF. Consequently, this study focuses on three crucial biomarkers, namely DRIL, HRFs, and cystoid spaces. These biomarkers were chosen due to their significance in determining the severity of DME and their distinctive characteristics that are easily identifiable in OCT images.

#### 3.1.1. Disorganization of Retinal Inner Layers (DRIL)

**Figure 3:**
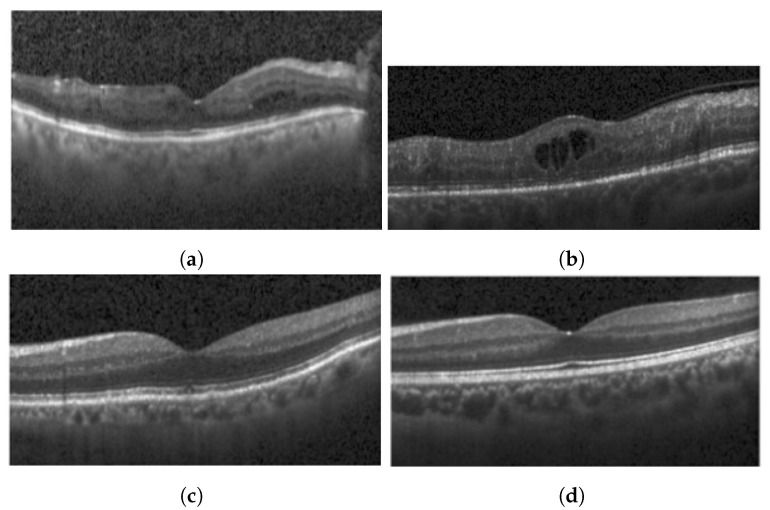
Typical OCT B-Scan images: (**a**,**b**) with DRIL (**c**,**d**) Normal.

**Figure 4:**
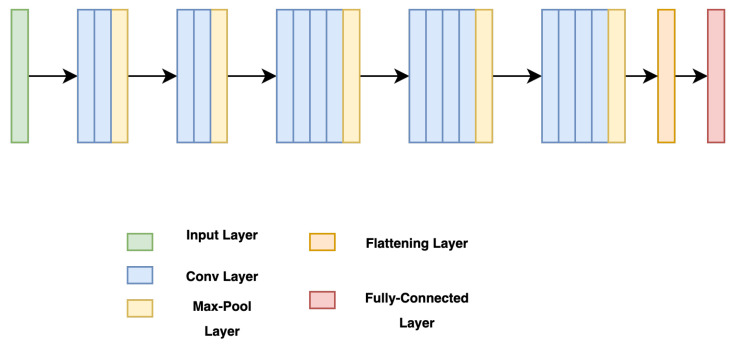
VGG-19 Network [27].

This study utilizes an OCT B-Scan image to examine the presence of DRIL. DRIL refers to a condition where the distinction between different inner retinal layers becomes unclear. Figure 3 showcases sample OCT B-Scan images with and without DRIL. Prior to feeding into the classification network, the images are resized to 416 × 416 and converted to grayscale. Moreover, each pixel value is scaled by a factor of 1/255, thereby limiting each pixel value to a range between 0 and 1. For classification, a VGG-19 [27] network (see Figure 4) is utilized as the backbone, with its weights pre-trained on the ImageNet dataset. VGG-19, consisting of 19 layers, is a dependable backbone network that requires less training time than VGG-16. This is due to the additional three convolutional layers in VGG-19 as compared to VGG-16, which allow the model to be robust even with a smaller sample size. Therefore, the network requires fewer samples than VGG-16, which ultimately reduces the training time. VGG-19 includes convolutional layers with a filter size of 3×3 and 1×1, a stride of 1 px, and a max-pooling layer of size 2×2 with a stride of 2. Following this, the feature map generated after the last max-pooling layer is flattened and fed into a fully connected layer, determining the final probability of the image belonging to either the DRIL or Normal class. All hidden layers utilize ReLU, except for the final layer, which uses a Softmax activation function. The model was trained using an Adam optimizer for 75 epochs.

#### 3.1.2. Hyperreflective Focii (HRF)

**Figure 5:**
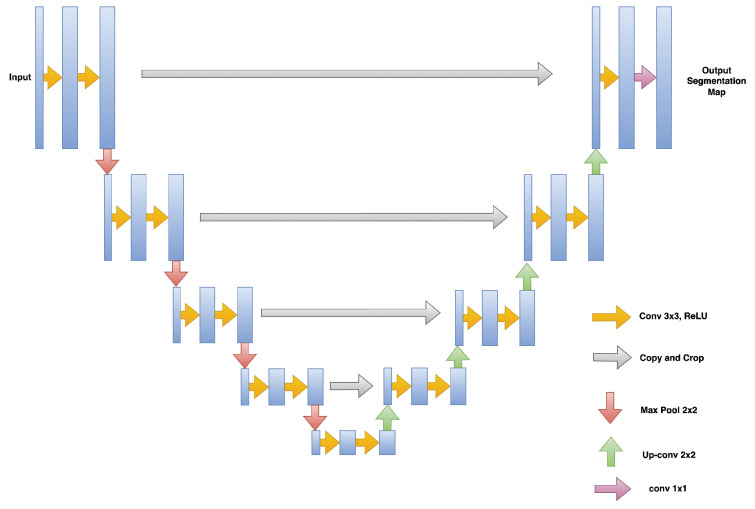
U-Net network architecture [28].

**Figure 6:**
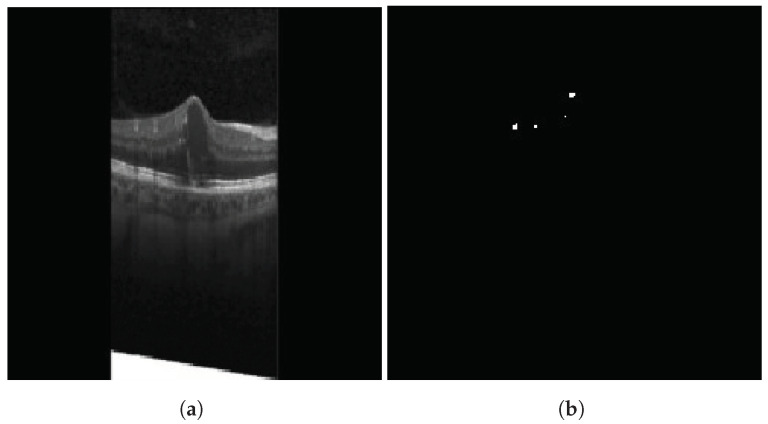
Result of HRF segmentation: (**a**) original image (**b**) segmentation map.

HRFs need to be segmented from the OCT B-scan image. Prior to input into the segmentation network, all images are resized to 192×192, with the three channels (RGB) preserved. A custom U-Net based model architecture [28] was employed for the purpose of semantic segmentation on OCT images, resulting in a segmented image mask containing the HRFs. U-Net comprises two paths: contraction and expansion. The contraction phase involves passing the image through a standard convolutional layer followed by a max-pool layer. A 3×3 filter is employed in each convolutional layer, with a rectified linear unit (ReLU) serving as the non-linear function. For pooling, a 2×2 filter with a stride of 2 is implemented. Following the contraction phase, the expansion phase is undertaken. The feature map generated after contraction is passed through an up-sampling layer followed by a 2×2 convolutional layer. The output from the up-sampling and feature extraction via the convolutional layer is then concatenated with the corresponding feature map from the contraction phase. A diagrammatic representation of a U-Net is depicted in Figure 5. The result of HRF segmentation for a sample OCT B-scan image is shown in Figure 6.

The custom U-Net model utilized in this study employs Binary Cross Entropy (BCE) Loss and dice similarity coefficient (DSC) for training the segmentation model [9]. The respective loss function is presented in Equation (1), wherein BCE is defined by Equation (2) and DSC is defined by Equation (3).

(1) $$\mathrm{Custom}\;\mathrm{Loss}=0.3*BCE\left(y_{i},p\left(y_{i}\right)\right)-DSC\left(y_{i},p\left(y_{i}\right)\right)$$

(2) $$\mathrm{BCE}=-(y_{i}*log\left(p\left(y_{i}\right)\right)+\left(1-y\right)log\left(1-p\left(y_{i}\right)\right))$$

(3) DSC=(2∗AreaofOverlap)/(TotalNumberofPixelsinBothImages)

where Area of Overlap = Area of overlap in the segmented class between the predicted segmentation map and the ground truth The value of the DSC ranges between 0 and 1. IOU can also be used as a similarity measure, which is the intersection between the union of the predicted segmentation map and the ground truth. It is positively correlated to the DSC.

#### 3.1.3. Cystoid Space

**Figure 7:**
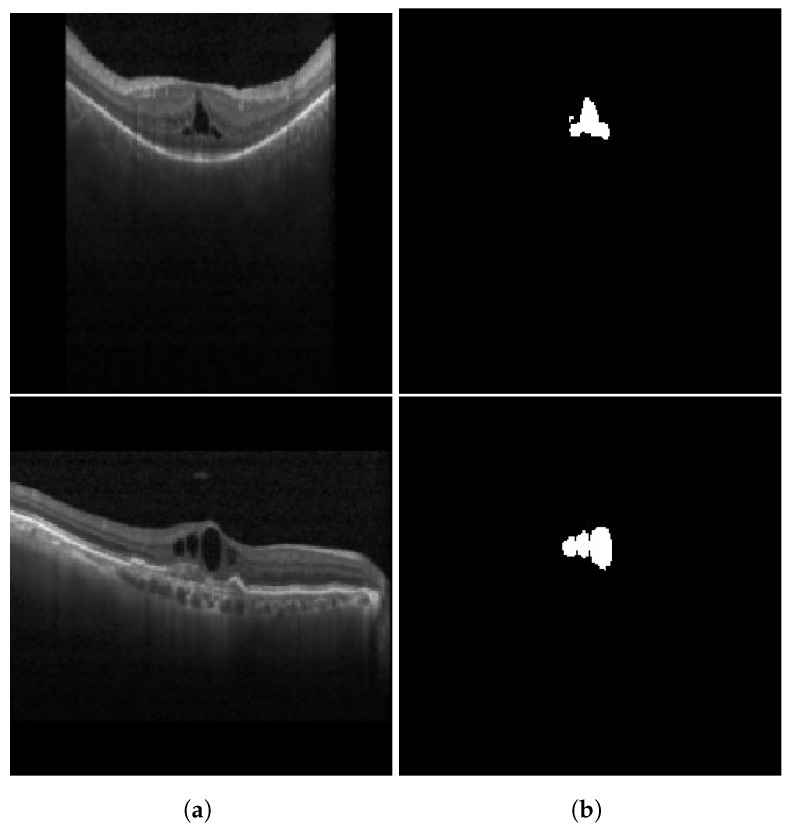
Results of cystoid segmentation: (**a**) original images (**b**) segmentation maps.

Similar to HRFs, cystoid spaces also require segmentation from the OCT B-scan image. Each image retains its three channels (RGB) and is resized to a 192×192 resolution. To remove any unwanted noise, a Gaussian blur with a kernel size of 5×5 is applied to the input image before it is passed to the segmentation network. For segmentation, the identical U-Net model utilized for HRF segmentation is employed for the segmentation of cystoid spaces from the OCT images. The outcome of the cystoid segmentation for a sample OCT B-scan image is displayed in Figure 7. In the current study, the custom U-Net model employs BCE Loss and DSC to train the segmentation model [9]. The loss function is given by Equation (1), where the DSC is defined as in Equation (3), and BCE is expressed as in Equation (2).

### 3.2. Generating Insights from Detected Biomarkers

Following the detection and segmentation of required biomarkers, the subsequent step is to extract from them features that indicate the severity of DME. Such features include the determination of whether DRIL is central, the position of HRFs, and the count and area of the cystoid spaces. Each of these is discussed in subsequent sections.

#### 3.2.1. Center Identification for DRIL

**Figure 8:**
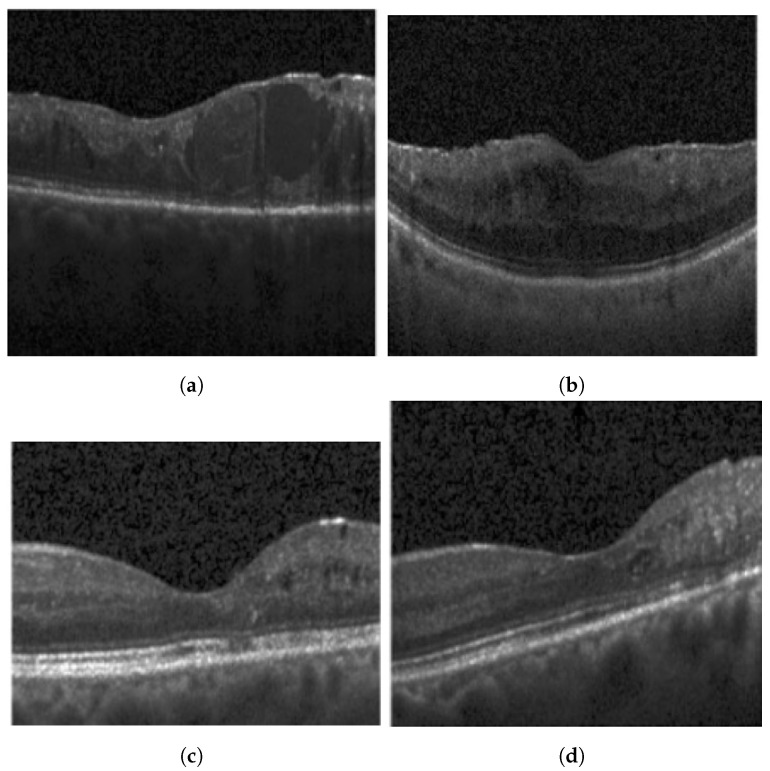
Results of DRIL image: (**a**,**b**) central DRIL. (**c**,**d**) d non- central DRIL.

DRIL is characterized as the inability to detect separation in INL, OPL, and the GCL-IPL complex on an OCT image [1]. Sun et al. [29] posited that DRIL with a horizontal extent exceeding 0.5 mm in the 1 mm central foveal area corresponds to worsened VA. Consequently, if DRIL is present in the OCT image, it becomes necessary to classify its position as either central or non-central. Figure 8 illustrates sample images for both central and non-central DRIL situations.

In classifying images with DRIL as either central or non-central, an object detection model, inspired by YOLOv5 [30,31], was first utilized to detect and draw bounding boxes around the 1 mm central foveal area. The decision to utilize a Yolov5-like model was influenced by its compact size and faster object detection runtime. Following the detection of the 1 mm foveal center, the VGG-19 network [27], similar to its previous application, was employed for classifying images as either DRIL or Normal. The weights utilized were pretrained on the ImageNet dataset, and the obtained feature map was flattened and fed into a fully connected layer. The model underwent training for 50 epochs with an Adam optimizer.

#### 3.2.2. Fetching the Location of HRFs

**Figure 9:**
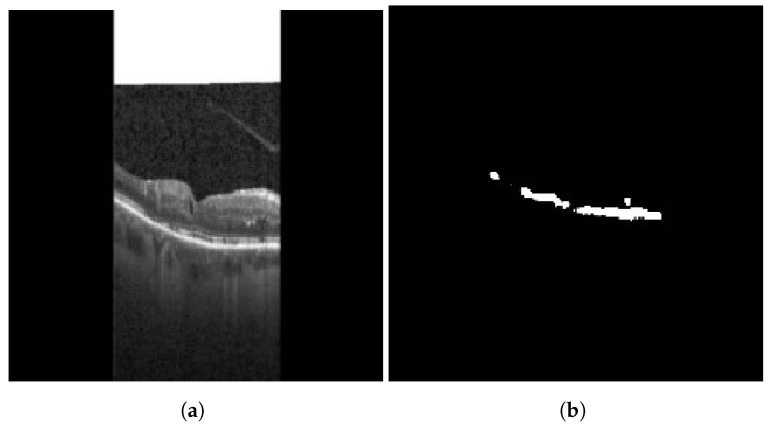
Results of OPL layer segmentation: (**a**) original image (**b**) segmentation map.

HRFs located in the OPL and ONL have a substantial negative impact on patients’ VA [32]. Therefore, following the segmentation of HRFs, those located above, within, or below the OPL layer require counting in the post-processing phase. The presence of a larger number of HRFs below or within the OPL layer indicates more severe DME than the former. This process is executed in two stages. The first stage involves the segmentation of the OPL from the OCT image. To achieve this, the same model utilized earlier for HRF segmentation is applied. The second stage involves determining the location of HRFs relative to the segmented OPL layer. Here, the position of HRFs is obtained from the segmented map resulting from HRF segmentation. This is done using OpenCV, which identifies HRFs as contours on the segmented map. The position of these contours is retrieved, providing the position of the HRFs. Ultimately, the position of the HRFs is compared with the OPL layer obtained from the other segmentation map (the map segmenting the OPL layer). Figure 9 illustrates the results of OPL layer segmentation for a sample OCT image. A comparison of these elements helps determine whether the HRF is present above the OPL layer or not. For instance, let (x1, y1) represent the position of an HRF. Then, in the segmentation map of OPL segmentation, for line x = x1, the value of the y-axis of the upper boundary of the OPL layer is examined to see whether it is greater than or less than y1. If it is greater than y1, the HRF is located inside or below the OPL layer. Otherwise, it is located above the OPL layer and, therefore, does not have as severe an impact as the former.

#### 3.2.3. Fetching of Cystoid Space Parameters

Yalçın et al. [33] outlined various parameters relating to the cystoid space in OCT images, indicative of the severity of DME. Four conclusions can be derived from their study: (i) There is a 58% probability of VA being less than 20/60 in eyes with a horizontal cyst diameter of ≥450 µm. (ii) There is a 73% probability of VA being greater than or equal to 20/60 in eyes with a horizontal cyst diameter of less than 450 µm. (iii) There is a 62% probability of VA being less than 20/60 in eyes with a vertical cyst diameter of ≥300 µm. (iv) There is a 69% probability of VA being greater than or equal to 20/60 in eyes with a vertical cyst diameter of less than 300 µm.

Apart from the relationship between cystoid diameter and VA, Nagai et al. [34] demonstrated that the number and area of cystoids present in the OCT image decreased with DME treatment, therefore signaling an improvement in disease severity. To incorporate these findings into our study and construct a computer vision-based model to grade DME severity based on cystoid spaces, cystoids must be segmented out from the OCT images. Subsequently, the count, area, and diameter (both horizontal and vertical) of these cystoids must be obtained. These parameters, together with the insights from recent studies [33,34], can then be used to determine a patient’s DME severity. After applying the U-Net model to OCT B-Scans to segment cystoids, the resulting segmentation map is utilized to extract information about each cystoid’s size and both horizontal and vertical diameters. For this study, OpenCV was used for this purpose. Initially, cystoids are detected as contours on the segmentation map. The count of these contours and the area of each contour are then obtained using OpenCV’s built-in functions. To determine the horizontal and vertical diameters of the cystoid, a minimum bounding rectangle is drawn around each detected contour. The dimensions of the rectangle represent the horizontal and vertical diameters of each cystoid space.

### 3.3. Fuzzy Rules

The utilization of fuzzy logic for tackling a variety of healthcare issues has been reported in several recent studies. For instance, Soltani et al. [23] employed a fuzzy logic approach for detecting glaucoma, attaining an impressive accuracy of 96.15%. Sibiya and Sumbwanyambe [24] similarly utilized a fuzzy system to identify maize common rust disease, demonstrating a test accuracy of 89%. Moreover, Jindal et al. [25] introduced a fuzzy logic method for diagnosing renal cancer, with their system yielding an accuracy of 96%, a sensitivity of 95.5%, a specificity of 96.1%, and a precision of 95.8%.

The next phase in this process, following the generation of insights from the biomarkers, involves applying fuzzy rules to these insights to determine the severity of DME. The identification and feature extraction outputs from each biomarker (DRIL, HRF, and cystoid spaces) are processed through the fuzzy engines. Subsequently, by adhering to the rules highlighted in our study (refer Table 1), the final DME severity is discerned, along with supplementary information that may aid the patient or physician in deciding upon a treatment plan. In the following subsections, we delve deeper into various fuzzy rules underpinned by recent research in this domain.

#### 3.3.1. Rules for Insights Generated on DRIL

In the research conducted by Endo et al. [26], pivotal correlations were discovered between factors obtained from OCT images and patients’ VA. The study determined that VA was significantly affected by factors such as the extent of DRIL, FAZ circularity, and EZ disruption. Notably, a direct proportionality was identified between the lengths of DRIL and EZ, while an inverse proportionality was seen between the lengths of DRIL and FAZ circularity. According to Grewal and Jaffe [1], DRIL emerges as a robust imaging biomarker for predicting VA in patients afflicted with DME. Furthermore, as highlighted by Sun et al. [29], VA can be adversely affected if the horizontal extent of DRIL is greater than 0.5 mm within the central foveal area of 1 mm. It is also worth noting that an increase in the extent of DRIL over a period of eight months correlates with a deteriorating VA for the same duration.

#### 3.3.2. Rules for Insights Generated on HRF

In the study conducted by Arthi et al. [2], an exploration of factors associated with HRF such as SRF and cystoid spaces was undertaken. This study established high positive correlations between these factors, suggesting HRF as a potential source of inflammation in patients. Similarly, Bolz et al. [32] demonstrated that the presence of HRF on the borders of the outer nuclear and the OPL leads to more light deflection than usual, consequently having an adverse effect on VA.

#### 3.3.3. Rules for Insights Generated on Cystoid Spaces

Yalçın et al. [33] indicated that (i) 58% probability of VA less than 20/60 is present in eyes with horizontal cyst diameter ≥ 450 and (ii) 73% probability of VA greater than or equal to 20/60 in eyes having horizontal cyst diameter amounting to less than 450 µm (iii) 62% probability of VA less than 20/60 in eyes with vertical cyst diameter ≥ 300 µm and (iv) 69% probability of VA greater than or equal to 20/60 in eyes with vertical cyst diameter < 300 µm. Nagai et al. [34] observed that the number and area of cystoid spaces decreased with DME treatment.

#### 3.3.4. Other Inferences

Dexamethasone (DEX) implant, a technique utilized for mitigating the impact on VA in patients with DME, has shown to improve DRIL [37]. As shown by Zur et al. [37], DRIL could be enhanced with a DEX implant, ameliorating DRIL by 60.6% and 75.0% at the intersection of Ganglion Cell and Inner Plexiform Layers (GCIPL) and INL at the intervals of four and twelve months, respectively. On the other hand, DRIL was reduced by 31.3% and 35.1% at the boundary between the INL and OPL after four and twelve months. Further, Zur et al. [37] also proposed that eyes devoid of HRF and DRIL and having sub-macular fluid are more likely to respond positively to DEX implants.

### 3.4. Combining It All

The input to the proposed model is an OCT B-Scan image affected by DME. Firstly, the necessary biomarkers for this study, namely DRIL, HRF, and cystoid spaces, are identified in the image. The DRIL is detected using a classifier, whereas the HRF and cystoid spaces are detected based on segmentation. Following this, the features that form part of the rules used in the fuzzy engine are extracted from these identified biomarkers. The resulting values are then passed through a fuzzy inference engine, where they are initially converted into fuzzy values through fuzzification. Subsequently, according to the rule base, the image is classified as representing severe, moderate, or mild DME.

## 4. Results

### 4.1. Dataset

**Figure 10:**
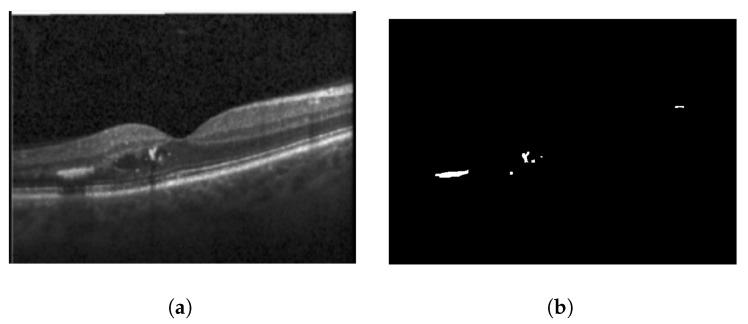
Sample images: (**a**) input, and (**b**) soft map from HRF segmentation mask.

**Figure 11:**
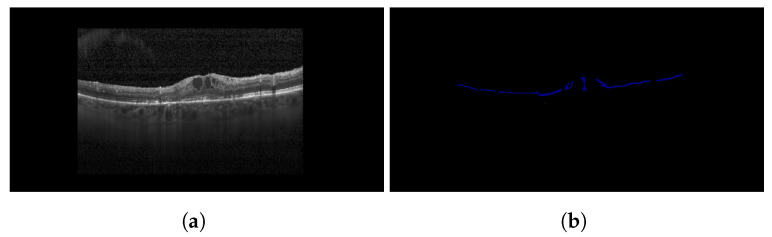
Sample images: (**a**) input, and (**b**) soft map from OPL layer segmentation mask.

**Figure 12:**
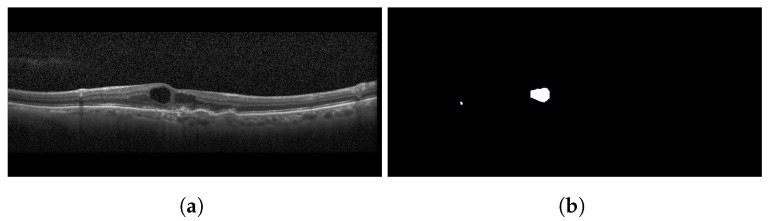
Sample images: (**a**) input, and (**b**) soft map from cystoid segmentation mask.

For this study, a custom dataset was curated and validated by an expert ophthalmologist. The dataset was created from approximately 150 high-resolution, low-noise images selected from a larger set of 1000 OCT B-Scan images with DME sourced from Kaggle [38,39,40]. All images were in JPEG format with a resolution of 416×416 pixels. The dataset was divided into five distinct categories corresponding to various tasks: DRIL/Normal classification, Central/non-Central DRIL, HRF segmentation, OPL layer segmentation, and cystoid segmentation. For the DRIL/Normal classification task, images with DME were further subdivided based on the presence of DRIL. 100 images (50 with DRIL and 50 non-DRIL images) were used for training and 30 for testing. Images with DRIL were further classified into central or non-central DRIL for the Central/non-Central DRIL classification model. This classification task utilized 100 images for training and 30 for testing. The categorization of these images was verified by an ophthalmologist. For the task of HRF segmentation, 30 annotated images of 192×192 pixels resolution served as the ground truth to train the model. Annotations were manually conducted using online software and verified by the ophthalmologist. Sample annotated images used for training the HRF segmentation model are illustrated in Figure 10. The patient’s OCT B-scan image was the model input, and the corresponding soft map, generated post-annotation, was considered the ground truth for model training. Similarly, for OPL layer segmentation, 22 images of 192×192 pixels resolution were used for training and five for testing. This task aided in determining the positions of HRFs, as shown in Figure 11. For the cystoid segmentation model, a separate data folder containing input OCT B-scan images and their corresponding soft maps, with annotated cystoid spaces, was created. The model was trained on 25 images (192×192 pixels resolution) and tested on 10 images. Sample annotated images utilized for training the cystoid segmentation model can be found in Figure 12.

### 4.2. Evaluation Metrics

Different metrics were used for classification and segmentation tasks. The DSC is used for segmentation to measure the model’s performance. It is given by Equation (3). For classification, standard validation metrics were employed, including sensitivity (also known as recall or True Positive Rate (TPR)), precision, $F_{1}$-score, and accuracy. These metrics predominantly evaluate the performance of true positives (TP), false positives (FP), false negatives (FN), and true negatives (TN) identified by the classification model. Sensitivity, the ratio of TP to the total actual positive cases and is given by Equation (4). A higher sensitivity value indicates that the model is good at capturing positive instances. Precision (refer Equation (5)) is the ratio of TP to all the cases predicted as positive. A higher precision value indicates that the model is good at ensuring that its positive predictions are indeed positive. $F_{1}$-score (refer Equation (6)) is the harmonic mean of precision and recall, and the system is considered better as this value increases. This metric is more informative than the standard accuracy score as it considers TP, FP, TN, and FN. Accuracy (refer Equation (7)) is the proportion of all predictions that are correct. This metric provides a general measure of the model’s performance across all classes.

(4) $$\mathrm{Sensitivity}=\frac{TP}{TP+FN}$$

(5) $$\mathrm{Precision}=\frac{TP}{TP+FP}$$

(6) $$F_{1}\text{-}\mathrm{score}=2\cdot \frac{\mathrm{Precision}\cdot \mathrm{Sensitivity}}{\mathrm{Precision}+\mathrm{Sensitivity}}$$

(7) $$\mathrm{Accuracy}=\frac{TP+TN}{TP+FP+FN+TN}$$

### 4.3. Biomarker Study Comparison

This study is the first to build classification models for DRIL/Normal and Central/non-Central DRIL and segmentation models for the OPL layer and cystoid spaces. There is a study, however, that has built a model for HRF segmentation. This has been discussed in detail, along with the individual performances of models for each biomarker, in the subsections below.

#### 4.3.1. DRIL

**Figure 13:**
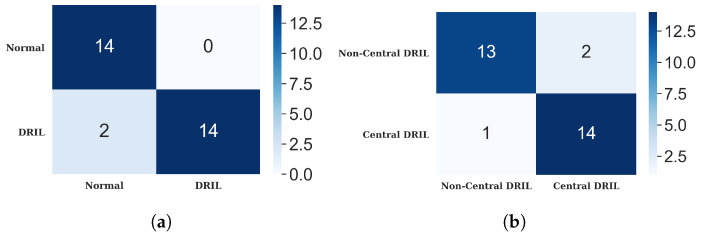
Confusion matrices for classification models (**a**) DRIL/normal (**b**) central/non-central DRIL.

**Figure 14:**
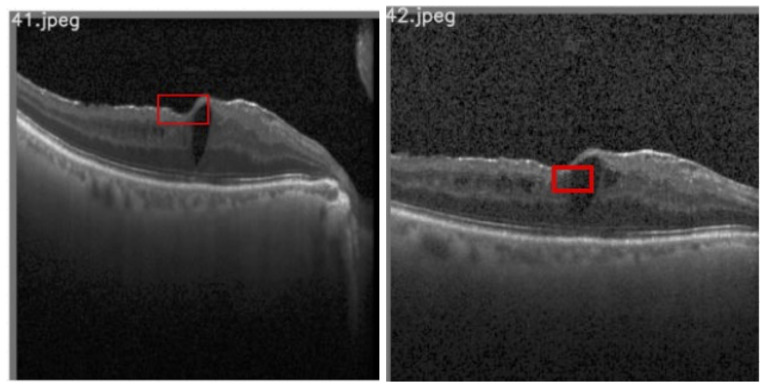
Results of object detection model. The red box indicates the detected central foveal area.

This study employed two classification models. The first model was used to distinguish between OCT images demonstrating the presence of DRIL and those without DRIL. Following this DRIL/Non-DRIL classification, a custom object detection model was utilized to pinpoint a 1 mm central foveal area. This was accomplished with a precision of 0.874, a recall of 0.636, and a mean average precision of 0.653. Subsequently, an additional classification model was used to categorize the images displaying DRIL as either Central or Non-Central DRIL. The confusion matrices of the DRIL/Non-DRIL classification and the Central/Non-Central DRIL are presented in Figure 13. The outcomes of the classification models are tabulated in Table 2. Sample images and their corresponding detection results are exhibited in Figure 14.

#### 4.3.2. HRF

This study uses two segmentation models for HRF detection and their positions: HRF segmentation and OPL layer segmentation. The result is shown in Table 3.

#### 4.3.3. Cystoids

For cystoids, the proposed segmentation model is utilized for detection. The result of the segmentation is given in Table 4.

### 4.4. Fuzzy Rules Quantification

Table 5 shows the results after applying the fuzzy engine to the features extracted from three biomarkers: DRIL, HRFs, and cystoids.

## 5. Discussion

Recently, fuzzy engines have been employed for the detection of various diseases. However, this system has not yet been applied to the detection of DME or detection of the severity of DME. Our study seeks to detect the severity of DME using a fuzzy engine. This study divides the entire methodology into different stand-alone models, each of which can be individually enhanced without affecting other models. For example, the biomarker extraction is one such model, where the inclusion of more biomarkers can potentially improve the overall model performance. Similarly, diverse features can be extracted from a biomarker, or alternative rules can be applied to each biomarker to enhance model performance. Such a modular solution facilitates further refinement of the model’s results by integrating additional biomarker extraction models, more descriptive features for each biomarker, and more precise rules for the features derived from each biomarker.

It has been observed that a DRIL exceeding 0.5 mm of horizontal length in a 1 mm central foveal area leads to a greater loss of VA [33]. However, minimal research has been conducted in predicting the presence of DRIL and its location in the central foveal area. Our model addresses this by initially classifying OCT B-Scan images into those with DRIL and those without. The model employs a VGG-19 network for image classification, followed by a fully-connected layer that generates the final probability of the image containing DRIL. The DRIL/Normal image classifier attained an accuracy of 93.33%. A comparison of the VGG-19 network with other backbone networks for DRIL/Normal image classification is presented in Table 6.

When DRIL is detected, a subsequent classifier determines if DRIL is located in the 1 mm central foveal area, thereby extending its overall performance and applicability. This network also employs a VGG-19 network, which achieved an accuracy of 93.00%. A comparison of the VGG-19 network with other backbone networks for Central/non-Central DRIL images is presented in Table 7.

Cystoid spaces are the second biomarker, which the model segments out from the OCT B-scan images and calculates their area, vertical diameter, and horizontal diameter and count before feeding the values into the fuzzy engine. Liu et al. [21] built an algorithm for CME segmentation using omnidirectional wave operators on OCT images and produced a DSC of 81.1% and a recall of 75.0%. Venhuizen et al. [22] developed a deep learning algorithm to segment intraretinal cystoid fluid from SD-OCT images with a DSC of 75.4%. Our model uses a custom U-Net-based architecture to segment the cystoid spaces and achieves a DSC of 95.07%. Table 8 draws a comparative study between segmentation models used for cystoid segmentation in previous studies and our model.

The final biomarker considered in this study for the detection of DME severity is HRF. These must be segmented from the OCT B-Scan image, and their positions relative to the OPL, either above or below, must be determined. Schlegl et al. [19] proposed an enhanced ResUnet model to segment HRFs from OCT B-scan images, achieving DSC of 65.26% and 63.49% on the Cirrus and Spectralis OCT datasets respectively. Xie et al. [20] proposed a segmentation-based model to detect HRF in SD-OCT images. It provided a DSC of 70.73%, a precision of 72.68%, and a recall of 68.89% using U-Net and image enhancement techniques. Our model outperforms these by achieving a DSC of 91.30% (refer Table 9). The increase in DSC can be attributed to changes in the HRF segmentation methodology and the architecture of the employed U-Net model. Contrary to the model proposed by Xie et al. [20], where the input SD-OCT image was denoised and enhanced before feeding into the U-Net as a 2-channel input, our model directly inputs the denoised image into the U-Net model. During encoding, our model consists of pairs of convolutional and up-sampling layers with intervening max-pooling layers, followed by a dropout layer after each convolutional layer to control model overfitting. For decoding, the standard U-Net architecture is followed, with concatenation of convolutional layers with the corresponding up-sampled layer. The final layer is a convolutional layer with one channel and a sigmoid function, yielding a segmentation map of size 192×192. To ascertain whether the HRFs are above or below the OPL layer, another segmentation model is trained to segment the OPL layer. We utilized the same network architecture as the other segmentation models, and segmented the OPL layer from the OCT B-Scan image with a DSC of 98.28%. Upon obtaining the segmentation map with the OPL layer and another map with segmented HRFs, these maps are overlaid to determine the relative position of HRFs to the OPL layer. Xie et al. [20] utilized 33 SD-OCT cubes from 27 patients, whereas Schlegl et al. [19] utilized 145 OCT images from private hospitals. This study leverages 30 publicly accessible OCT images manually annotated with online tools and validated by ophthalmologists.

Following the classification of OCT B-Scan images based on the presence and position of DRIL, segmenting HRFs and cystoids, and extracting features from each biomarker—which include categorizations of DRIL or Normal, Central or Non-Central DRIL, the position of HRFs relative to the OPL, and the count, area, and vertical and horizontal diameters of cystoids—these features are input into a fuzzy logic system. The rules for this system are informed by insights from existing studies examining the relationships between each biomarker and DME. Consequently, the fuzzy engine outputs the predicted severity of DME. This study contributes to clinical practice by enabling physicians to estimate the severity of DME using solely a patient’s OCT B-Scan image. This could assist in determining appropriate treatment approaches and monitoring disease progression in response to therapeutic interventions. Future research could explore the generation of predictive OCT images for patients with DME, given their baseline OCT images. This could offer valuable insights into the potential progression of the disease, aiding clinicians in their treatment strategies and enhancing the likelihood of patient recovery.

## 6. Conclusions

In this study, we proposed a comprehensive model to grade the severity of DME from OCT B-Scan images by focusing on three key biomarkers: DRIL, HRF, and cystoid spaces. The methodology adopted in this work encompasses three stages. In the first stage, the image is classified as either Normal or DRIL, and further, DRIL images are subclassified into Central DRIL or Non-Central DRIL. Simultaneously, the HRF and cystoid spaces are segmented from the OCT image. The second stage involves the extraction of relevant features from the segmented images, predominantly using OpenCV. The extracted features are then passed into a fuzzy logic engine during the final stage. This engine, guided by rules derived from recent studies in this domain, outputs the ultimate DME severity grade.

The outcomes of this study can equip healthcare professionals with a valuable tool to grade DME severity, thereby facilitating personalized, targeted treatment plans for patients. While the current model presents promising results, it could potentially benefit from incorporating a broader range of biomarkers for severity evaluation, or by integrating more rules into the fuzzy engine. Such expansions, of course, would require further analysis based on previous research findings and expert medical input for validation. In this way, our model’s reliability and applicability could be further enhanced in future studies.

## Figures and Tables

**Table 1:** Rules used to develop the fuzzy engine.

Biomarker	Rule	Study Supporting the Rule
DRIL	If DRIL of >0.5 mm in a central foveal area of >1 mm then it represents lower VA.	[29]
	Patients with no DRIL and no HRF respond better to dexamethasone (DEX) implants.	[35]
HRF	If the HRF is present on the borders of outer nuclear and the outer plexiform layer, or below it, then it considers deflecting more light than usual hence worsening VA.	[32]
	HRF is more implies SRF is also a high chance, and if SRF is there, it means more fluid in the eye hence implying higher severity of DME.	[32]
Cystoid Space	The high number, and area of cystoids, predict worse VA.	[34]
	73% probability of VA ≥ 20/60 is present with eyes having horizontal cyst diameter <450 µm.	[33]
	69% probability of VA ≥ 20/60 is present with eyes having vertical cyst diameter <300 µm.	[33]
	Patients with cystoids of size greater than >450 um horizontal diameter and >300 um vertical diameter have a high chance of BCVA of <20/60, in which case they should be advised not to drive and read.	[36]
	If the horizontal diameter of the cystoid is <450 um and the vertical diameter <300 um, then the patient has a high probability of responding to ranibizumab.	[36]

**Table 2:** Result of DRIL/normal and central/non-central DRIL classification models.

S.No	Classification Model	Training Size	Testing Size	Accuracy (%)	Sensitivity (%)	Specificity (%)	F1-Score (%)
1	DRIL/Normal	100	30	93.33	0.875	1.0	0.93333
2	Central/non-Central DRIL	100	30	90.00	0.9333	0.8666	0.9032

**Table 3:** Result of HRF segmentation, OPL layer segmentation.

S.No	HRF Segmentation	Training Size	Testing Size	DSC (%)
1	HRF segmentation	20	8	91.30
2	OPL layer segmentation	22	5	98.28

**Table 4:** Results of the cystoid segmentation model.

S.No	Segmentation Model	DSC (%)
1	Our Model	95.07

**Table 5:** Final results for sample OCT B-Scan images.

Image	DRIL	HRF	Cystoid	Final Score
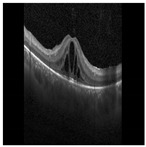	Yes, Central	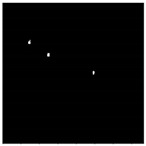	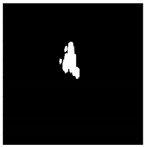	Severe
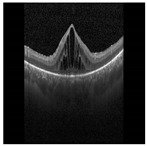	Yes, Central	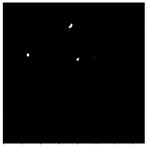	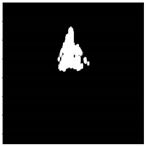	Severe
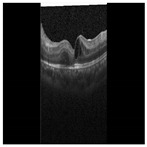	Yes, Non-central	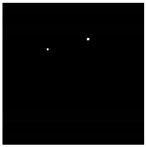	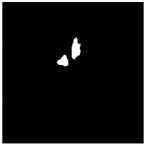	Medium
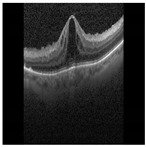	Yes, Central	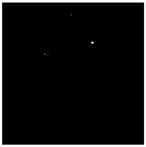	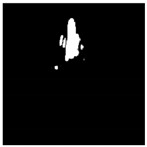	Severe
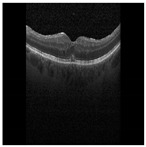	No	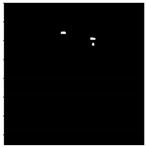	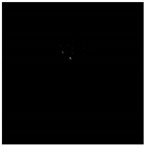	Mild
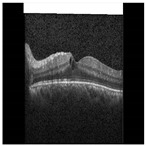	Yes, Central	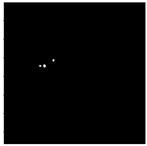	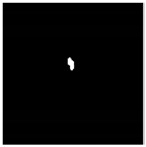	Mild
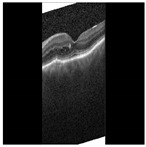	Yes, Non-central	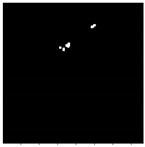	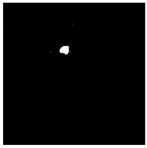	Mild

**Table 6:** Results of VGG-19 network and other backbone networks on DRIL/Normal classification.

S.No	Backbone Network	Training Accuracy (%)	Validation Accuracy (%)
**1**	VGG19	97.96	70.0
2	ResNet50	93.88	60.0
3	ResNet101	96.94	50.0
4	Inception	97.96	63.33

**Table 7:** Results of VGG-19 network and other backbone networks on Central/non-Central DRIL classification.

S.No	Backbone Network	Training Accuracy (%)	Validation Accuracy (%)
1	VGG19	98.47	60.0
2	ResNet50	95.83	40.0
3	ResNet101	83.33	50.0
4	Inception	87.50	40.0

**Table 8:** Comparison of different cystoid segmentation models.

S.No	Segmentation Model	DSC (%)
1	Using omnidirectional wave operator [21]	81.1
2	Deep Learning-based Algorithm [22]	75.4
**3**	Our Model	95.07

**Table 9:** Comparison between models for HRF segmentation.

S.No	Segmentation Model	DSC (%)
1	U-Net with Image Enhancement [20]	70.73
2	ResUnet+ (Cirrus) [19]	65.23
3	ResUnet+ (Spectralis) [19]	63.49
**4**	Proposed Model	91.30

## Data Availability

The data that support the findings of this study are openly available at https://data.mendeley.com/datasets/rscbjbr9sj/2 (accessed on 19 August 2021). The annotations and ground truth labels are available from the corresponding authors on request.
